# Alfalfa saponins inhibit oxidative stress-induced cell apoptosis through the MAPK signaling pathway

**DOI:** 10.1080/13510002.2021.2017681

**Published:** 2021-12-21

**Authors:** Yalei Cui, Fen Li, Xiaoyan Zhu, Junying Xu, Abaidullah Muhammad, Yanyan Chen, Defeng Li, Boshuai Liu, Chengzhang Wang, Zhichang Wang, Sen Ma, Xule Liu, Yinghua Shi

**Affiliations:** a College of Animal Science and Technology, Henan Agricultural University, Zhengzhou, People’s Republic of China; b Henan Key Laboratory of Innovation and Utilization of Grassland Resources, Zhengzhou, People’s Republic of China; c Henan Herbage Engineering Technology Research Center, Zhengzhou, People’s Republic of China

**Keywords:** Alfalfa saponins, oxidative stress, piglets’small intestinal epithelial cell, antioxidant enzymes, cell viability, cell apoptosis, MAPK signaling pathway

## Abstract

**Background:**

Oxidative stress could seriously affect the growth performance of piglets. As natural extracts of *Alfalfa (Medicago sativa)*, alfalfa saponins have been shown to function as antioxidants in piglets *in vivo*. However, few studies have investigated the effects and mechanism of alfalfa saponins against oxidative stress in piglet cells *in vitro*. In the current study, piglets’ small intestinal epithelial cell line (IPEC-J2) was explored to investigate the protective effects of alfalfa saponins on injured cells induced by H_2_O_2_.

**Methods:**

To investigate the effects and mechanism of alfalfa saponins against oxidative stress in piglet cells, the cell viability, activity of antioxidant enzymes, LDH and the amount of MDA were detected in H_2_O_2_-treated cells after the cells were pre-incubated with alfalfa saponins. The mechanism of alfalfa saponins against H_2_O_2_-induced oxidative cell damage was explored by detecting the expression of mitochondrial apoptosis-related proteins. Furthermore, the signaling pathway of alfalfa saponins in IPEC-J2 cells under oxidative stress was also investigated.

**Results:**

The results indicated that alfalfa saponins could rescue cell viability, elevate the activity of antioxidant enzymes and down-regulate the activity of LDH and the amount of MDA in H_2_O_2_-induced cells.

**Conclusion:**

Alfalfa saponins could inhibit oxidative stress-induced cell mitochondrial apoptosis through the MAPK signaling pathway, thereby providing a new method for improving antioxidant stress ability by means of nutritional regulation.

## Introduction

*Alfalfa (Medicago sativa)* is the most economical forage legume cultivated globally, and belongs to the leguminous family [1]. It is rich in protein, vitamins, minerals and other important nutrients [2,3]. The rich nutrients contain many potential active ingredients, such as saponins, flavonoids and polysaccharides [3]. Saponins are natural extracts with unique biological activities and function as antioxidants [4,5]. In animals, if the accumulation of free radicals exceeds the scavenging ability of the anti-oxidation defense system, then peroxidation damage of tissue cells would occur [6]. Oxidative stress can cause DNA oxidative damage and abnormal protein expression, causing toxic effects on cells [7,8]. Many studies have also proved that the oxidative stress caused by reactive oxygen species is the key link of cell apoptosis [9–11]. Oxidative stress is a common state of weaned piglets, which makes piglets suffer great economic loss [12]. Therefore, alfalfa saponins have attracted widespread attention. Previous research has indicated that alfalfa saponins could increase the activity of antioxidant enzymes in weaned piglets [13]. But the mechanism of alfalfa saponins against oxidative stress in piglet cells has not yet been investigated.

Intestinal mucosal epithelial cells are important functional cells of the intestine which play a key role in digestive enzymes’ secretion, nutrients’ digestion, absorption, transport, stress response and immune barrier [14,15]. The previous study has shown the antioxidant effects of alfalfa saponins on rat intestinal epithelial cells (IEC-6 cell line) [16]. However, IEC-6 is a cell line from rat, one kind of model animal, whose digestive tube construction is very different from that of swine [17]. Moreover, different types and characteristics of cells have different tolerances to H_2_O_2_ [18]. Our previous study in piglets has demonstrated that alfalfa saponins showed antioxidant effects *in vivo* [13]. To further investigate whether alfalfa saponins also play an antioxidant role in piglet cells *in vitro*, IPEC-J2, a type of non-tumorigenic epithelial cell line isolated from neonatal piglet mid-jejunum, was exploited in this study to investigate the protective effects further and regulatory mechanism of alfalfa saponins on the oxidative stress-induced apoptotic piglet cells, providing a new method for improving antioxidant stress ability of piglets by means of nutritional regulation.

## Materials and methods

### Culture of small intestinal epithelial cells (IPEC-J2) of piglets

IPEC-J2 was donated by Henan Provincial Key Research Laboratory of Animal Food Safety of Henan Agricultural University. It was cultured by DMEM-F12 (containing amino acids, vitamins, inorganic salts, 10% FBS and other components, Gibco, Australia) in a 5% CO_2_ incubator at 37°C.

### Cell viability assay

Alfalfa saponins were extracted from the leaves and stem of alfalfa with 62% purity, containing compounds of Daidzein, Hederin, Soyasaponin, Ruscogenin, etc., provided by Hebei Bao’en Biotechnology Co., Ltd (Shijiazhuang, China). To screen the suitable concentration of alfalfa saponins, IPEC-J2 cells (1.2 × 10^4^ cells/well) were inoculated on 96-well plates (Corning, USA) for 24 h. The cells were divided into 6 treatment groups. The concentrations of alfalfa saponins were 0, 100, 200, 400, 500, 600 and 800 μg/mL, respectively. The cells were cultured in an incubator at 37°C and 5% CO_2_ for 24 h, and then an MTT solution (Solarbio, Beijing, China) was added, gently mixed and incubated for 4 h in a CO_2_ incubator. The medium was carefully aspirated from each well. 100 μL of Formazan Solubilization Solution (Solarbio, Beijing, China) was added to each well. The 96-well plate was put on a shaker to mix gently for 10 min to dissolve the formazan crystals. The absorbance value of each well was measured at 490 nm with a microplate reader (Thermo Scientific, USA). The absorbance value indicated the cell viability.

### Effects of H_2_O_2_ on the viability of IPEC-J2 cells

1.2 × 10^4^ cells/well were seeded into 96-well plates. After being cultured for 24 h, cells were divided into 5 groups with different concentrations of H_2_O_2,_ such as 0, 200, 300, 400, and 500 μmol/L H_2_O_2_. After being cultured at 37°C and 5% CO_2_ for 24 h, the MTT solution was added and incubated for 4 h. The cell viability was detected at 490 nm with a microplate reader (Thermo Scientific, USA), and then the optimal concentration of H_2_O_2_ was selected.

### Effects of alfalfa saponins on the viability of IPEC-J2 cells induced by H_2_O_2_

To determine the protective concentration of alfalfa saponins on H_2_O_2_-induced IPEC-J2 cells, 1.2 × 10^4^ cells/well were inoculated in 96 well. After 24 h of culture, different concentrations of 0, 100, 200, 300 and 400 μg/mL alfalfa saponins were added in IPEC-12 cells for 24 h, and then 300 μmol/L H_2_O_2_ was added for 24 h. After adding the MTT solution for 4 h, the absorbance of each well was measured at 490 nm with a microplate reader (Thermo Scientific, USA).

### Intracellular SOD, GSH-Px, CAT, LDH and MDA were determined

3 × 10^5^ IPEC-J2 cells were inoculated in a 6-well plate. After 24 h of culture, the cells were divided into four groups: (1) Negative Control: cells without treatment; (2) 200 μg/mL alfalfa saponins group: 200 μg/mL alfalfa saponin solution was added to the cell medium for 24 h. Then, the medium was replaced with a new cell medium and the cells were being cultured for 24 h; (3) H_2_O_2_ group: the medium was replaced with a new cell medium, after cultured for 24 h, 300 μmol/L H_2_O_2_ was added and was being cultured for 24 h; (4) 200 μg/mL alfalfa saponins + H_2_O_2_ group: 200 μg/mL alfalfa saponin solution was added to the cell medium for 24 h. Then the cell medium was removed and 300 μmol/L H_2_O_2_ was added for 24 h.

Cells were collected and a motor-operated grinder was used to grind cells in an ice-water bath. Then a BCA kit (Beyotime) was used to measure protein concentration in the sample. The activities of superoxide dismutase (SOD), glutathione peroxidase (GSH-PX), catalase (CAT), lactate dehydrogenase (LDH) and the amount of malondialdehyde (MDA) were determined following Nanjing Jiancheng Kit. The principles of methods used to measure the activities of these enzymes are described below. As O_2_^-^ can reduce WST1 to form WST1-Formazan, which has absorption at 450 nm and SOD can remove O_2_^-^, thus inhibiting the formation of WST1-Formazan. GSH-PX can promote the reaction of H_2_O_2_ and GSH to generate H_2_O and GSSG; GSH-PX activity can be represented by its enzymatic reaction rate; GSH reacts with dithio-dinitrobenzoic acid to produce 5-thio-dinitrobenzoic acid anions with absorbance at 412 nm; GSH consumption caused by a non-enzymatic reaction must be deducted when calculating the activity of this enzyme. The decomposition of H_2_O_2_ by CAT can be quickly halted by adding ammonium molybdate; the remaining H_2_O_2_ reacts with ammonium molybdate to produce a yellowish complex with absorbance at 405 nm. LDH catalyzes lactic acid to pyruvate; pyruvate reacts with 2,4-dinitrophenylhydrazine to generate a compound with light chocolate color; then, the activity of LDH can be determined at 450 nm. MDA can be combined with thiobarbituric acid (TBA) to form red compounds with a maximum absorption peak at 532 nm.

### Annexin V assay of apoptosis

Cells (5 × 10^4^/well) were plated onto a 24-well plate, and cells were divided into 4 groups, as described above. Apoptosis assay of IPEC-J2 cells using annexin V (Invitrogen) was performed according to the manufacturer’s protocol. Cells were washed twice using PBS and resuspended in a 195 μL Annexin V-FITC binding buffer. Subsequently, 5 μL Annexin V-FITC and 10 μL propidium iodide (PI) were added. The cells were incubated for 20 min in the dark. At last, the cells were analyzed by a fluorescent microscope.

### Western blot analysis

Cells from four groups were collected, and the BCA kit (Beyotime) was used to measure protein concentration (900 μg/mL) in the sample. SDS-PAGE was used to separate the proteins. After protein (54 μg) was transferred to a nitrocellulose membrane (Bio-Rad, USA), the membrane was blocked with 5% milk at room temperature for 1 h. Then the membrane was incubated with a primary antibody for 2 h at room temperature. Subsequently, the membrane was incubated with horseradish peroxidase (HRP)-conjugated goat anti-rabbit IgG (Sigma-Aldrich, USA) for 2 h. Proteins were detected using Western LightningTM Plus-ECL Oxidizing Reagent Plus (Perkin Elmer, USA). The antibodies of Bcl-2 (26 KD), caspase-3 (32/17 KD), caspase-9 (46 KD), Bax (21 KD) were purchased from PROTEINTECH GROUP, USA. The antibodies of p38 (40 KD), p-p38 (thr180/tyr182, 43 KD), ERK (44/42 KD), p-ERK (thr 202/tyr 204, 44/42 KD), JNK (46/54 KD), p-JNK (thr183/tyr185, 46/54 KD) were bought from Cell Signaling Technology, USA. The antibody of GAPDH (37 KD) was purchased from TransGen Biotech, Beijing, China.

### Statistical analysis

Data were analyzed using the SPSS 20.0, and the one-way analysis of variance (one-way ANOVA) technique was used to testify the overall significance of the data. Duncan’s method was used for multiple comparisons between groups if the difference was significant. All data were calculated as mean ± standard deviation ($\bar{x}$  ± s). *P* < 0.05 is a significant difference. *P* < 0.01 is an extremely significant difference.

## Results

### Non-cytotoxic concentration of alfalfa saponins in IPEC-J2 cells culture

**Figure 1.**
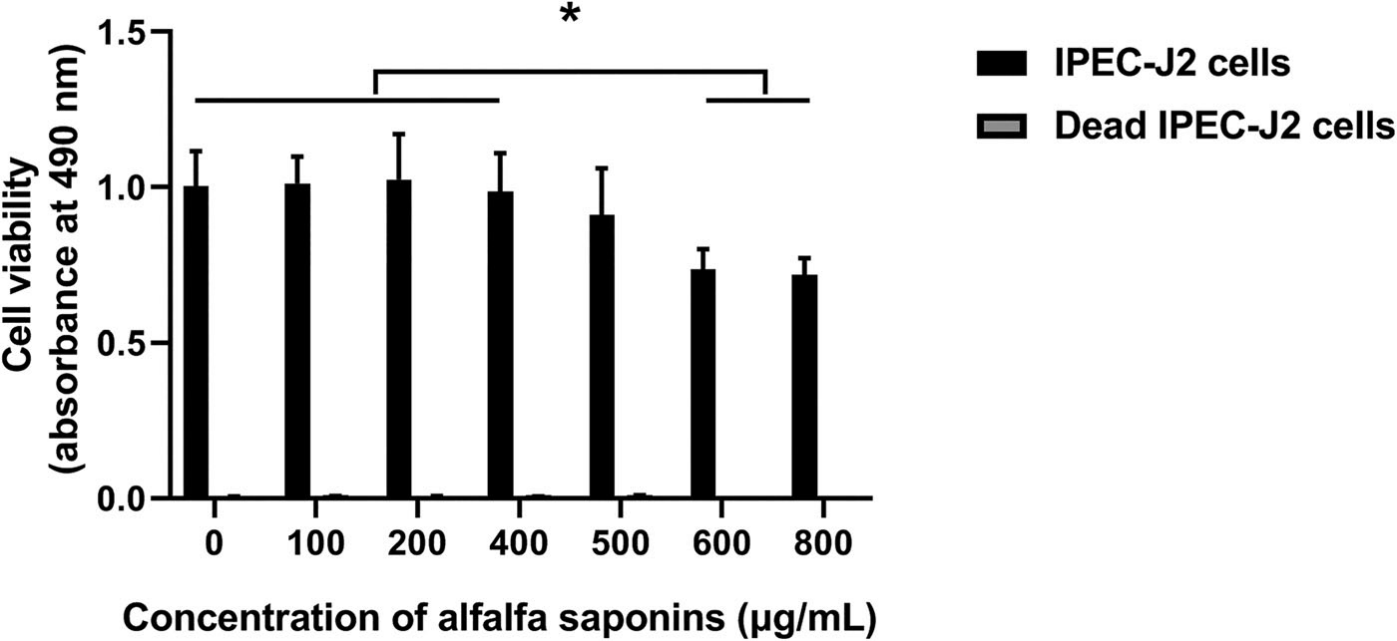
Effects of different concentrations of alfalfa saponins on the viability of IPEC-J2 cells. Dead cells were used as control. In all panels, statistically significant difference between treatments were represented with asterisks (*p < 0.05; **p < 0.01).

In order to explore the effects of alfalfa saponins on the cell viability of IPEC-J2 cells, the cell viability of IPEC-J2 cells under different concentrations of alfalfa saponins (100, 200, 400, 500, 600, 800 μg/mL) was determined. Different concentration gradients were used to evaluate the maximum non-cytotoxic concentration of alfalfa saponins on IPEC-J2 cell viability. The results showed that the viability of IPEC-J2 cells slightly decreased, but there was no significant difference between the group without alfalfa saponins and alfalfa saponin concentration from 100 to 500 μg/mL. However, the viability of IPEC-J2 cells significantly decreased (*P* < 0.05) when the concentrations of alfalfa saponins were 600 and 800 μg/mL (Figure 1). As antioxidants can reduce MTT directly in the absence of cell mitochondria activity, dead cells were used as a control group to understand the chemical reactivity of the alfalfa saponins with MTT. In dead cells, there was no significant difference when the final concentration of alfalfa saponins was 100–800 μg/mL, but 600 and 800 μg/mL alfalfa saponins could slightly reduce MTT.

### Oxidative stress state induced by H_2_O_2_ in IPEC-J2 cells

**Figure 2.**
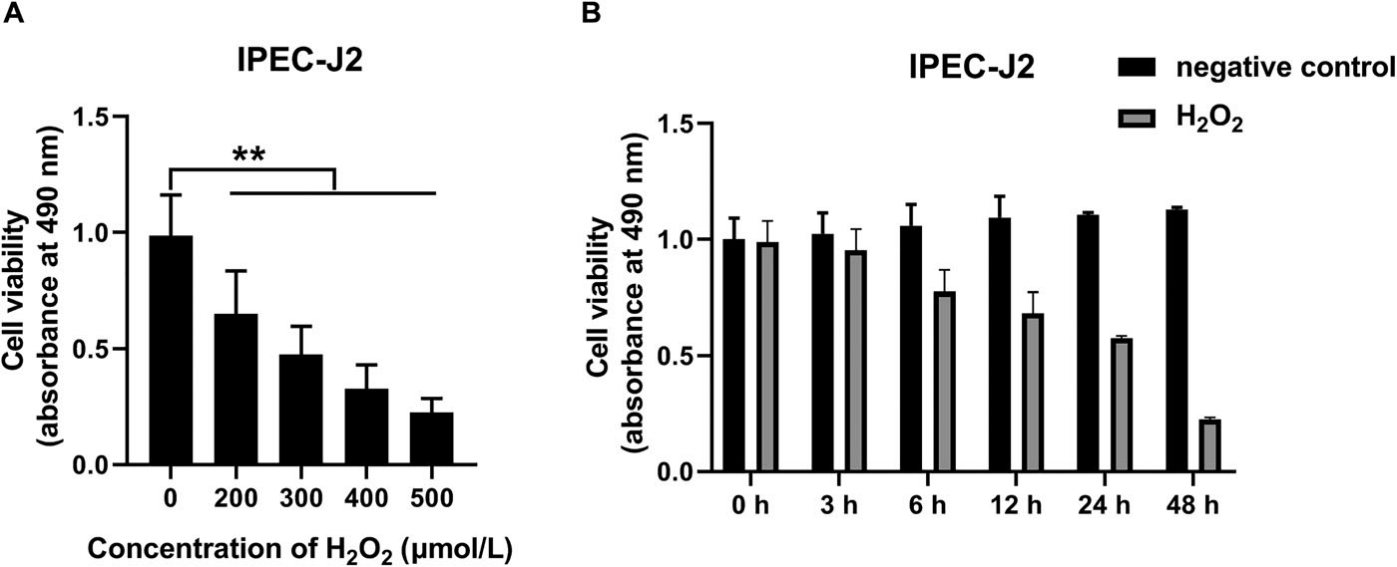
Effects of different concentrations and treatment times of H_2_O_2_ on the viability of IPEC-J2 cells: (A) effects of different concentrations of H_2_O_2_ on the viability of IPEC-J2 cells and (B) time-dependent effects of H_2_O_2_ on cell viability of IPEC-J2 cells. In all panels, statistically significant differences between treatments were represented with asterisks (*p < 0.05; **p < 0.01).

To establish the oxidative stress state in IPEC-J2 cells, the appropriate concentration and treatment time of H_2_O_2_ were determined, respectively. To determine the appropriate concentrations of H_2_O_2_ for IPEC-J2 oxidative stress stimulation, IPEC-J2 cells were treated with different concentrations of H_2_O_2_ (0, 200, 300, 400 and 500 μmol/L) for 24 h, the cell viability significantly decreased compared with the control group, which reduced to 66%, 48%, 33% and 23%, respectively (*P* < 0.01, Figure 2A). The 50% lethal concentration of H_2_O_2_ on the cell viability of IPEC-J2 cells was 300 μmol/L. Therefore, the treatment concentration of 300 μmol/L H_2_O_2_ was selected for the subsequent oxidative stress research. To determine the appropriate time for H_2_O_2_ to stimulate IPEC-J2 cells, different treatment times of 300 μmol/L H_2_O_2_ (0, 3, 6, 12, 24 and 48 h) were set to determine the cell viability of IPEC-J2 cells. In the control group without H_2_O_2_ treatment, there was no significant difference in cell viability during 48 h culture period (Figure 2B). However, after H_2_O_2_ treatment, the cell viability of IPEC-J2 cells at 3, 6, 12, 24 and 48 h was significantly lower than that of the control group at the same time point, which decreased to 94%, 74%, 64%, 53% and 20%, respectively (*P* < 0.01, Figure 2B). It can be seen that the 50% lethal treatment time of H_2_O_2_ on the survival rate of IPEC-J2 cells was 24 h. Therefore, the H_2_O_2_ oxidative stress treatment time in this study was 24 h.

### Effects of alfalfa saponins on the viability of H_2_O_2_-induced oxidative injury cells

**Figure 3.**
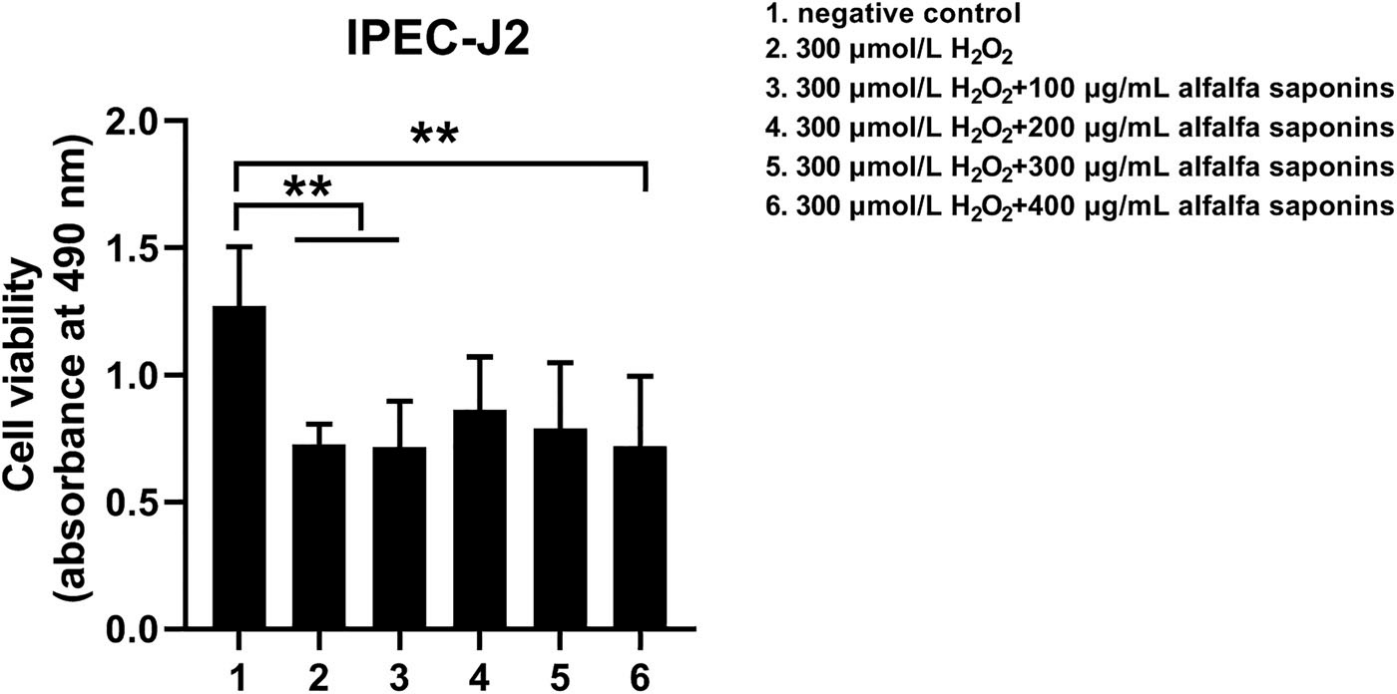
Effects of alfalfa saponins against H_2_O_2_-induced oxidative stress damage on IPEC-J2 cells’ viability. In all panels, statistically significant difference between treatments were represented with asterisks (*p < 0.05; **p < 0.01).

To ensure the protective effects of alfalfa saponins on IPEC-J2 cells, the concentration of alfalfa saponins was less than 500 μg/mL in subsequent experiments. The cell viability decreased significantly (*P* < 0.01) after being treated with H_2_O_2_, while it increased by the pre-treatment of 200 and 300 μg/mL alfalfa saponins (*P* < 0.01, Figure 3). Moreover, the cell viability in the pre-treatment group of 200 and 300 μg/mL alfalfa saponins showed no significant difference with the negative control group, indicating that pre-treatment of 200 and 300 μg/mL alfalfa saponins could rescue cell viability. 200 μg/mL alfalfa saponins pre-treated cells had better protective effects on H_2_O_2_-induced IPEC-J2 cells (Figure 3). Therefore, 200 μg/mL of alfalfa saponins was selected as the suitable concentration for cell oxidative stress.

### The anti-oxidant activity of alfalfa saponins in oxidative damage cells

**Figure 4.**
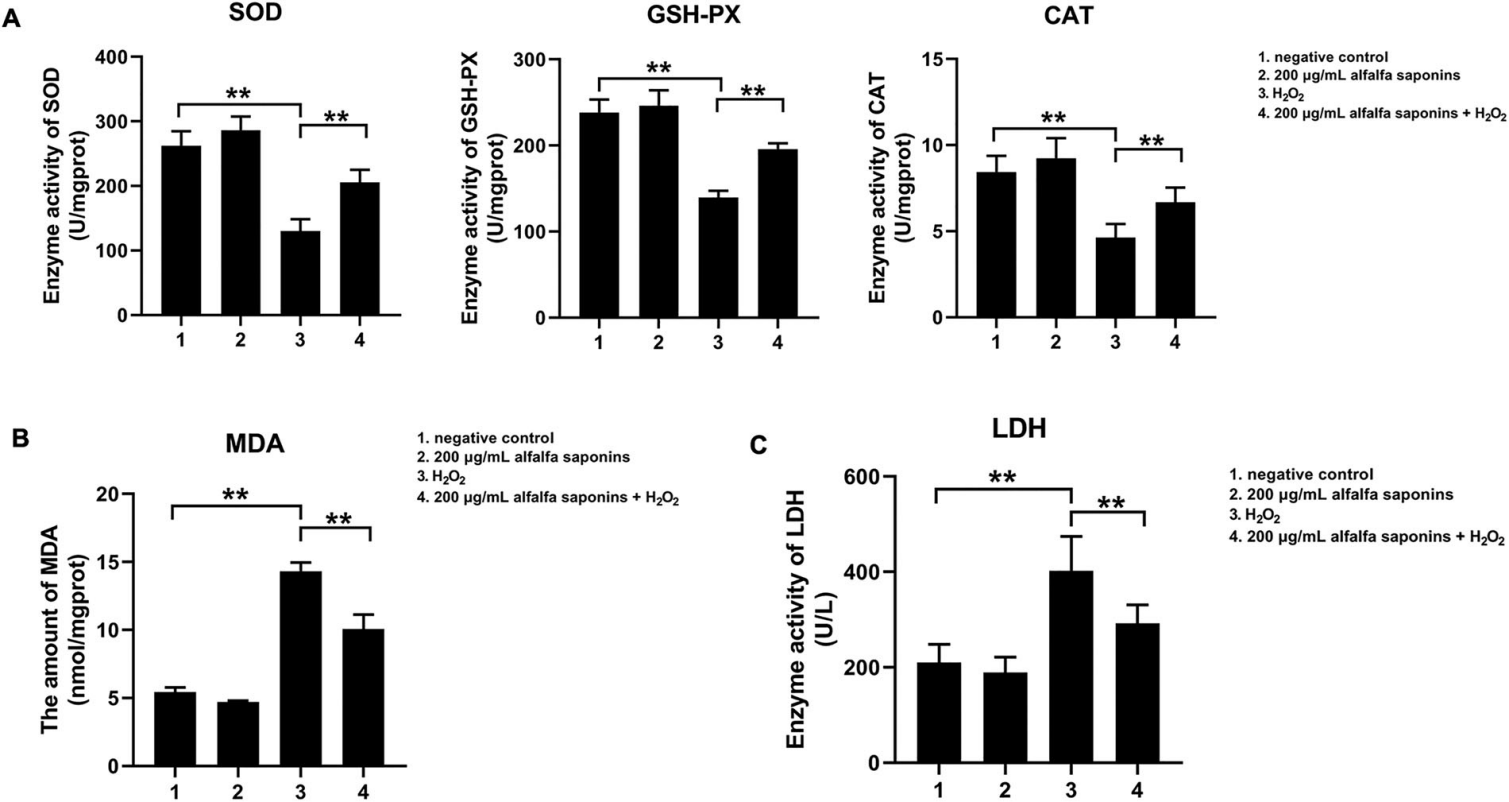
Effects of alfalfa saponins on the antioxidant system in IPEC-J2 cells. (A) Effects of alfalfa saponins on the antioxidant enzyme activity of IPEC-J2 cells induced by H_2_O_2_. When the SOD inhibition ratio reaches 50%, the corresponding enzyme amount is one SOD activity unit (U). (B) Effects of alfalfa saponins on the amount of MDA induced by H_2_O_2_ in IPEC-J2 cells. (C) Effects of alfalfa saponins against H_2_O_2_-induced LDH activity in IPEC-J2 cells. In all panels, statistically significant difference between treatments were represented with asterisks (*p < 0.05; **p < 0.01).

To explore the effects of alfalfa saponins on the antioxidant capacity of IPEC-J2 cells, the activities of intracellular superoxide dismutase (SOD), glutathione peroxidase (GSH-PX) and catalase (CAT) were detected. The results indicated that the activities of SOD, GSH-PX and CAT in oxidative stress cells were significantly lower than those of negative control cells (*P* < 0.01, Figure 4A). And the amount of MDA significantly increased (*P* < 0.01, Figure 4B). Compared with cells treated with H_2_O_2_, the activities of SOD, GSH-PX and CAT in the 200 μg/mL alfalfa saponins + H_2_O_2_ group increased significantly (*P* < 0.01, Figure 4A), and the amount of MDA decreased obviously (*P* < 0.01, Figure 4B). These findings demonstrated that alfalfa saponins could improve oxidation resistance. Compared with the control group, 200 μg/mL alfalfa saponins group had little effects on the release of LDH in IPEC-J2 cells, while H_2_O_2_ can result in a large amount of release of LDH, which increased by 92.3% compared with the control group (*P* < 0.01, Figure 4C). The amount of cellular LDH releases reduced by 27.6% in 200 μg/mL alfalfa saponins + H_2_O_2_ group compared with cells treated with H_2_O_2_ (*P* < 0.01, Figure 4C). These results indicated that alfalfa saponins could reduce the release of LDH in H_2_O_2_-induced oxidative damage cells.

### Regulation of downstream pathway of H_2_O_2_-induced cell apoptosis by alfalfa saponins

**Figure 5.**
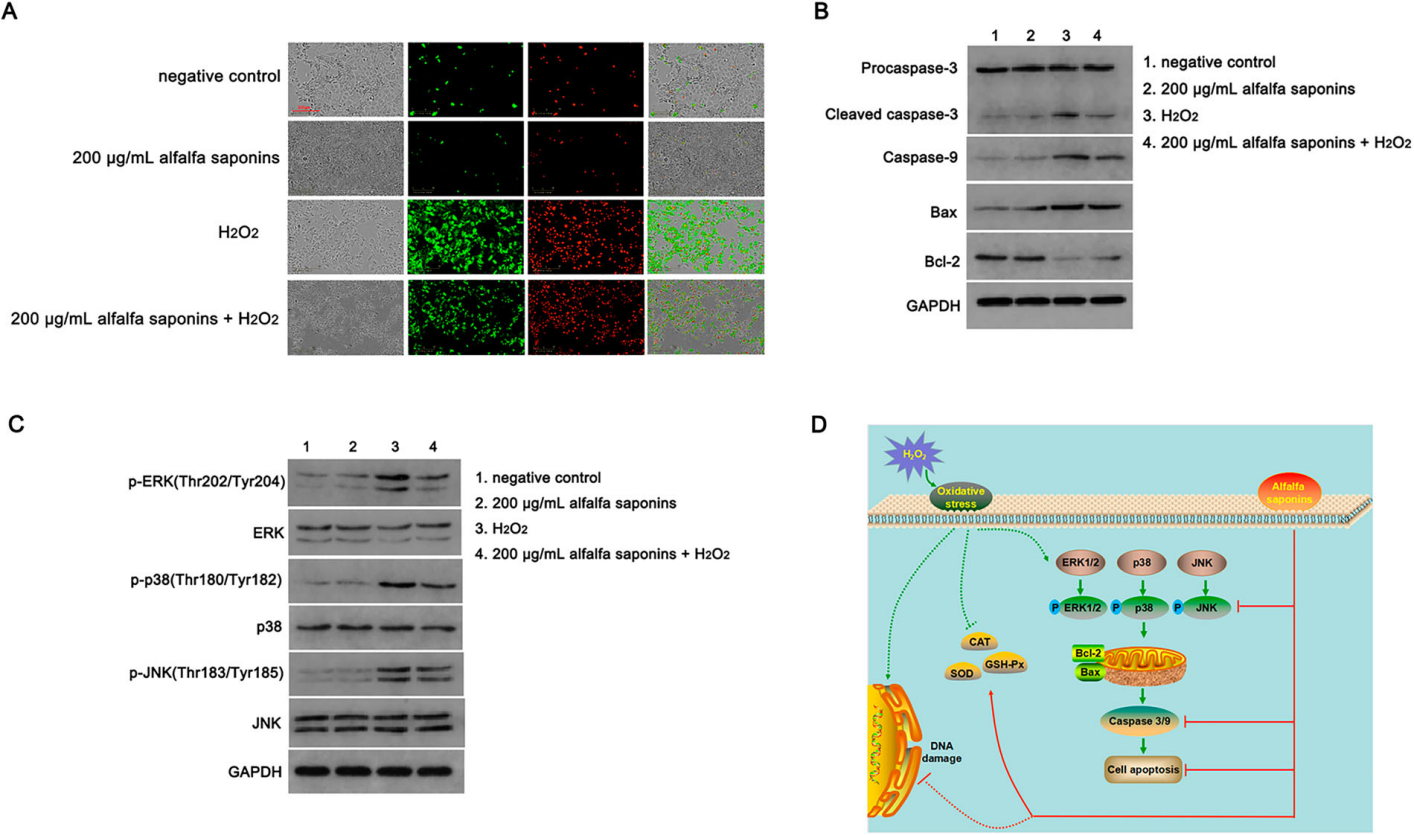
Cell apoptosis analysis of IPEC-J2 cells. (A) Annexin V/PI staining was used to detect cell apoptosis ratio. The green fluorescence of Annexin V-FITC represented by early apoptotic cells. The red fluorescence of PI staining indicated necrotic cells and apoptotic cells in the middle and late stages. The yellow color indicated that Annexin V-FITC and PI could stain the cells. (B) The expression level of apoptosis-related proteins was detected by western blot. (C) Effects of alfalfa saponins on MAPK pathway in IPEC-J2 cells. (D) Protective mechanism of alfalfa saponins in oxidative stress cells.

To investigate the antioxidant mechanism of alfalfa saponins on oxidative stress induced by H_2_O_2_ in IPEC-J2 cells, Annexin V-FITC&PI double staining was used to analyze the apoptosis ratio of cells. The apoptosis of IPEC-J2 cells treated with H_2_O_2_ was significantly higher than that of cells without H_2_O_2_ treatment (Figure 5A). There was no significant difference in the apoptosis of the 200 μg/mL alfalfa saponins group than that of the control group. However, the apoptosis ratio of the H_2_O_2_-treated group was significantly increased. Compared with the H_2_O_2_-treated group, the apoptosis ratio of cells in the 200 μg/mL alfalfa saponins + H_2_O_2_ group was significantly decreased (Figure 5A). To further determine the mechanism of alfalfa saponins’ regulation of H_2_O_2_-induced apoptosis in IPEC-J2 cells, the expression levels of caspase-3, caspase-9, Bcl-2 and Bax in cells were determined by western blot. There was no significant difference in the expression level of procaspase-3 among all groups (Figure 5B). Compared with the control group, the expression levels of cleaved-caspase-3, caspase-9, Bax and Bcl-2 in the 200 μg/mL alfalfa saponins group were not significantly different. The expression of cleaved caspase-3, caspase-9 and Bax in the H_2_O_2_-treated group significantly increased compared to the control group, while the expression of Bcl-2 significantly decreased. The intracellular cleaved caspase-3, caspase-9 and Bax have significantly decreased in the 200 μg/mL alfalfa saponins + H_2_O_2_ group, and the expression of Bcl-2 was up-regulated while comparing with the H_2_O_2_-treated group. These findings indicated that alfalfa saponins could up-regulate the expression of anti-apoptotic gene Bcl-2 and down-regulate the expressions of pro-apoptotic cleaved-caspase-3, caspase-9 and Bax, thus playing a protective role in oxidative IPEC-J2 cells (Figure 5B).

To study the signaling mechanism of alfalfa saponins in the antioxidant protection of IPEC-J2 cells, the protein expressions of three MAPK signaling pathways, including cellular ERK1/2, JNK and p38, were detected. There was no significant difference in the total protein expression of ERK1/2, p38 and JNK among the four groups. The phosphorylation levels of ERK1/2, p38 and JNK in the H_2_O_2_-treated group were significantly increased than those of the control group (*P* < 0.01, Figure 5C), while the phosphorylation levels of the three proteins in the 200 μg/mL alfalfa saponins + H_2_O_2_ group were effectively decreased compared with the H_2_O_2_-treated group. The results showed that alfalfa saponins could inhibit the activation of the MAPK signaling pathway induced by H_2_O_2_ in IPEC-J2 cells thus having protective effects on IPEC-J2 cells.

Overall, alfalfa saponin pre-treatment can inhibit mitochondrial apoptosis through the MAPK signaling pathway, thus achieving antioxidant activity and further reducing oxidative damage of piglet cells (Figure 5D).

## Discussion

### Effects of alfalfa saponins on the viability of IPEC-J2 cells

Recent studies mainly focus on the effects of alfalfa saponins on cell cholesterol. There are few studies on the effects of alfalfa saponins on the mechanism of oxidative stress [19–21]. To determine alfalfa saponins’ toxicity to IPEC-J2 cells and the optimal concentration range of alfalfa saponins in cells, MTT was used to detect the effects of alfalfa saponins on the viability of IPEC-J2 cells, which laid a foundation for the subsequent experiment. The results showed that the concentration of alfalfa saponins had no effects on the viability of IPEC-J2 cells when it was 0–500 μg/mL. However, when the final concentration of alfalfa saponins was 600 and 800 μg/mL, the viability of IPEC-J2 cells significantly decreased, indicating that too much alfalfa saponins had toxicity on cells.

### Effects of different concentrations and times of H_2_O_2_ on the viability of IPEC-J2 cells

In general, H_2_O_2_ is often used in the modeling of oxidative stress [22,23]. It was found that H_2_O_2_ had oxidative toxicity on IPEC-J2 cells in this study. Concentration-dependent and time-dependent assays were conducted to determine the cell viability of cells decreased by about half after the treatment with 300 μmol/L H_2_O_2_ for 24 h compared with the control group. The results indicated that the median lethal concentration of H_2_O_2_ in IPEC-J2 cells was 300 μmol/L, which was higher than the median lethal concentration (150 μmol/L) of H_2_O_2_ in IEC-6 cells, indicating IPEC-J2 cells were much more tolerant to H_2_O_2_ than IEC-6 cells [16]. For the suitable concentration and appropriate time of IPEC-J2 oxidative stress stimulation, cells treated with 300 μmol/L H_2_O_2_ for 24 h were determined as the oxidative stress model of this experiment.

### Effects of alfalfa saponins on the viability of H_2_O_2_-induced oxidative injury in IPEC-J2 cells

In the case of oxidative stress in IPEC-J2 cells induced by H_2_O_2_, alfalfa saponins’ pre-treatment could increase the cell viability of IPEC-J2 cells, indicating that alfalfa saponins have a certain protective effect on the oxidative damage of IPEC-J2 cells. Moreover, 200 μg/mL alfalfa saponins have a better protective effect on the oxidative damage of IPEC-J2 cells, suggesting that alfalfa saponins also play an antioxidant role in piglet cells, but the demand was relatively large than that of IEC-6 cells [16].

### Effects of alfalfa saponins on antioxidant enzymes of H_2_O_2_-induced oxidative damage in IPEC-J2 cells

Superoxide dismutase (SOD) is an important superoxide anion (O_2_^–^) scavenger [24]. It is an enzyme that can protect the structural integrity and function of cell membrane. Glutathione peroxidase (GSH-PX) is the main antioxidant enzyme to remove H_2_O_2_ and some organic hydroperoxides in organism [25]. Catalase (CAT) is a kind of oxidase widely distributed in biological organisms, promoting the decomposition of H_2_O_2_ and thus protect cells from free radical damage [26]. SOD, GSH-PX and CAT are very important antioxidants in organisms. When cells are stimulated by outside, the antioxidant capacity could be improved by antioxidants, which can weaken the damage of free radicals to the cell membrane. Malondialdehyde (MDA) is the product of lipid peroxidation [27]. H_2_O_2_ treatment would increase the MDA amount of IPEC-J2 cells and reduce the antioxidant ability of SOD, GSH-Px and CAT, resulting in excessive reactive oxygen species [28,29]. Compared with the H_2_O_2_ group, the antioxidant ability of SOD, GSH-Px and CAT in the 200 μg/mL alfalfa saponins + H_2_O_2_ group significantly increased, while the amount of MDA decreased. Therefore, alfalfa saponins could alleviate the oxidative stress of IPEC-J2 cells by increasing the antioxidant ability of antioxidant enzymes in cells and reducing the amount of MDA. Lactate dehydrogenase (LDH) is a stable cytoplasmic enzyme that can be quickly released into the cell culture when the cell membrane is damaged. Therefore, it is often used as an indicator to reflect the degree of cell damage [30]. The results showed that when IPEC-J2 cells were subjected to H_2_O_2_ oxidative stress, the cell membrane was seriously damaged, and the activity of LDH was greatly increased. Alfalfa saponins’ pre-treatment could effectively alleviate the release of LDH.

### Effects of alfalfa saponins on H_2_O_2_-induced apoptosis of IPEC-J2 cells

It was found that cell apoptosis was closely related to free radicals produced by oxidative stress [31]. FITC–AnnexinV/PI fluorescence staining results of the current study suggested that the antioxidant effects of alfalfa saponins on H_2_O_2_-induced oxidative stress in IPEC-J2 cells might be realized by inhibiting cell apoptosis. Cell mitochondria-mediated apoptosis is an important mechanism of oxidative damage [32]. Caspase-3 is the executor of apoptosis and can be directly involved in the early initiation of apoptosis, apoptosis signal transduction and apoptosis late events. Stimulated by apoptosis signal, pro-caspase-3 forms an active form of caspase-3 in the activation process [33]. In addition, the anti-apoptotic gene (Bcl-2) and pro-apoptotic gene (Bax) in the Bcl-2 gene family are mainly involved in regulating apoptosis in the mitochondrial apoptotic pathway [34]. The results of this study showed that there was no difference in procaspase-3 expression among all groups. After H_2_O_2_ treatment, the expression of cleaved caspase-3, caspase-9 and Bax increased significantly, while the expression of Bcl-2 decreased significantly. 200 μg/mL alfalfa saponin pre-treatment decreased the expression of cleaved caspase-3, caspase-9 and Bax, and increased the expression of Bcl-2, which indicated that the mechanism of alfalfa saponins against H_2_O_2_-induced IPEC-J2 cell apoptosis might be achieved by up-regulating Bcl-2 and down-regulating the expression of cleaved caspase-3, caspase-9 and Bax, thus inhibiting mitochondrial apoptosis. MAPKs signal transduction pathway is mainly used to transduce extracellular stimulus signals into cells and nucleus and cause a series of physiological and biochemical reactions (such as cell proliferation, differentiation, metabolism and apoptosis) [35,36]. MAPKs are mainly composed of three subgroups of proteins, including ERK1/2, p38 and JNK. ERK1/2 signaling pathway plays an important role in growth factor-mediated cell proliferation and differentiation [37]. The activation of p38 and JNK pathways may be related to apoptosis and various pathological and physiological processes during stress [38]. Zhou et al. [39] reported that ERK1/2, JNK and p38 MAPK will be obviously activated when intestinal epithelial cells are damaged by oxidation. Moreover, the activation of these kinases will further promote the phosphorylation of related transcription factors and cytoplasmic proteins, leading to the occurrence of cell apoptosis [40]. This study found that the degree of phosphorylation of ERK1/2, p38 and JNK significantly increased after H_2_O_2_ treatment, while three protein phosphorylation levels declined obviously after the alfalfa saponins pre-treatment, showing that alfalfa saponins can inhibit the activation of ERK1/2, p38 and JNK in cells to achieve the effects of anti-apoptosis and anti-oxidative damage, thus playing a protective role in oxidative damage cells. The earlier study [16] has shown the antioxidant effects of alfalfa saponins on rat intestinal epithelial cells (IEC-6 cell line), but the justification for this current study is to demonstrate further that piglet cells (IPEC-J2 cell line) are much more tolerant to H_2_O_2_ than IEC-6 cells. Alfalfa saponins also play an antioxidant role in IPEC-J2, but the demand was relatively large than that of IEC-6 cells. Moreover, this study further verifies alfalfa saponins could inhibit oxidative stress-induced cell mitochondrial apoptosis through the MAPK signaling pathway in piglet cells *in vitro*, thereby providing a new method for improving antioxidant stress ability of piglets by exploiting alfalfa saponins.

## Data Availability

The authors declare that data supporting the findings of this study are available within the article.
